# Poly[(6-carboxy­picolinato-κ^3^ *O* ^2^,*N*,*O* ^6^)(μ_3_-pyridine-2,6-dicarboxyl­ato-κ^5^ *O* ^2^,*N*,*O* ^6^:*O* ^2′^:*O* ^6′^)dysprosium(III)]

**DOI:** 10.1107/S1600536809039075

**Published:** 2009-10-03

**Authors:** Xu Li, Qing-Yang Lian, Qiu-Hui Meng, Yi-Fan Luo, Rong-Hua Zeng

**Affiliations:** a School of Chemistry and Environment, South China Normal University, Guangzhou 510006, People’s Republic of China.; b South China Normal University, Key Laboratory of Technology of Electrochemical Energy Storage and Power Generation in Guangdong Universities, Guangzhou 510006, People’s Republic of China

## Abstract

In the title complex, [Dy(C_7_H_3_NO_4_)(C_7_H_4_NO_4_)]_*n*_, one of the ligands is fully deprotonated while the second has lost only one H atom. Each Dy^III^ ion is coordinated by six O atoms and two N atoms from two pyridine-2,6-dicarboxyl­ate and two 6-carboxy­picolinate ligands, displaying a bicapped trigonal-prismatic geometry. The average Dy—O bond distance is 2.40 Å, some 0.1Å longer than the corresponding Ho—O distance in the isotypic holmium complex. Adjacent Dy^III^ ions are linked by the pyridine-2,6-dicarboxyl­ate ligands, forming a layer in (100). These layers are further connected by π–π stacking inter­actions between neighboring pyridyl rings [centroid–centroid distance = 3.827 (3) Å] and C—H⋯O hydrogen-bonding inter­actions, assembling a three-dimensional supra­molecular network. Within each layer, there are other π–π stacking inter­actions between neighboring pyridyl rings [centroid–centroid distance = 3.501 (2) Å] and O—H⋯O and C—H⋯O hydrogen-bonding inter­actions, which further stabilize the structure.

## Related literature

For the isotypic holmium analogue, see: Fernandes *et al.* (2001 ▶). For other related structures, see: Hong (2007 ▶); Huang *et al.* (2008 ▶); Idrees *et al.* (2009 ▶); Rafizadeh & Amani (2006 ▶); Thallapally *et al.* (2008 ▶).
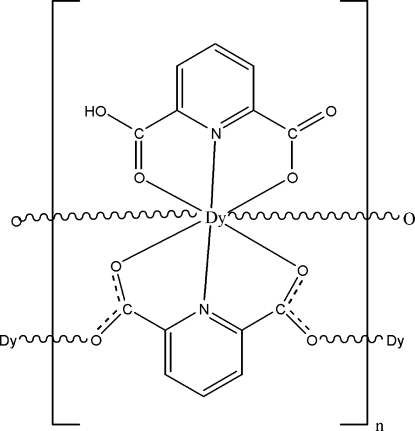


## Experimental

### 

#### Crystal data

[Dy(C_7_H_3_NO_4_)(C_7_H_4_NO_4_)]
*M* *_r_* = 493.72
Monoclinic, 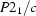

*a* = 12.2151 (14) Å
*b* = 8.3703 (10) Å
*c* = 13.4698 (16) Å
β = 102.332 (1)°
*V* = 1345.4 (3) Å^3^
*Z* = 4
Mo *K*α radiation
μ = 5.61 mm^−1^
*T* = 296 K
0.23 × 0.21 × 0.19 mm

#### Data collection

Bruker APEXII area-detector diffractometer
Absorption correction: multi-scan (*SADABS*; Bruker, 2004 ▶) *T*_min_ = 0.359, *T*_max_ = 0.415
6670 measured reflections
2413 independent reflections
2305 reflections with *I* > 2σ(*I*)
*R*_int_ = 0.024

#### Refinement

*R*[*F* ^2^ > 2σ(*F* ^2^)] = 0.021
*wR*(*F* ^2^) = 0.051
*S* = 1.12
2413 reflections
229 parameters
1 restraint
H atoms treated by a mixture of independent and constrained refinement
Δρ_max_ = 0.58 e Å^−3^
Δρ_min_ = −1.43 e Å^−3^

### 

Data collection: *APEX2* (Bruker, 2004 ▶); cell refinement: *SAINT* (Bruker, 2004 ▶); data reduction: *SAINT*; program(s) used to solve structure: *SHELXS97* (Sheldrick, 2008 ▶); program(s) used to refine structure: *SHELXL97* (Sheldrick, 2008 ▶); molecular graphics: *ORTEPIII* (Burnett & Johnson, 1996 ▶) and *PLATON* (Spek, 2009 ▶); software used to prepare material for publication: *SHELXL97*.

## Supplementary Material

Crystal structure: contains datablocks I, global. DOI: 10.1107/S1600536809039075/sj2654sup1.cif

Structure factors: contains datablocks I. DOI: 10.1107/S1600536809039075/sj2654Isup2.hkl

Additional supplementary materials:  crystallographic information; 3D view; checkCIF report

## Figures and Tables

**Table 1:** Selected bond lengths (Å)

Dy1—O8^i^	2.266 (2)
Dy1—O6^ii^	2.314 (3)
Dy1—O1	2.328 (2)
Dy1—O5	2.385 (3)
Dy1—O7	2.405 (2)
Dy1—N2	2.469 (3)
Dy1—N1	2.488 (3)
Dy1—O4	2.513 (2)

Symmetry codes: (i) 
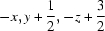
; (ii) 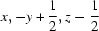.

**Table 2:** Hydrogen-bond geometry (Å, °)

*D*—H⋯*A*	*D*—H	H⋯*A*	*D*⋯*A*	*D*—H⋯*A*
O2—H2*A*⋯O4^iii^	0.811 (19)	1.73 (2)	2.518 (3)	164 (5)
O2—H2*A*⋯O3^iii^	0.811 (19)	2.65 (3)	3.303 (4)	139 (4)
C9—H9⋯O1^iv^	0.93	2.42	3.332 (4)	165
C1—H1⋯O5^v^	0.93	2.42	3.123 (5)	133
C2—H2⋯O3^iii^	0.93	2.47	3.393 (5)	174

Symmetry code: (iii) 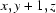; (iv) 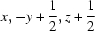; (v) 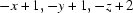.

**Fig. 1.**
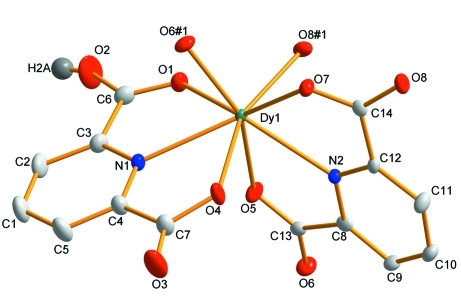
The molecular structure showing the atomic-numbering scheme, with displacement ellipsoids drawn at the 30% probability level. Symmetry codes: (#1) x, 0.5-y, -0.5+z; (#2) -x, 0.5+y, 1.5-z.

**Fig. 2.**
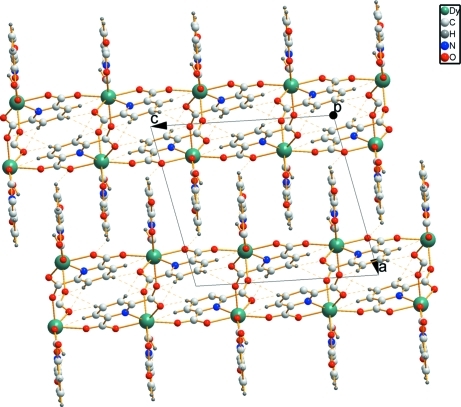
A view of the three-dimensional supramolecular network. Hydrogen bonds are shown as dashed lines.

## supplementary crystallographic information

### Comment

Research on the design and synthesis of metal-organic frameworks (MOFs) in recent years has become an active area in the fields of crystal engineering and supramolecular chemistry, not only because of their tremendous potential applications in gas storage, chemical separations, ion exchange, microelectronics, nonlinear optics, and heterogeneous catalysis, but also because of their intriguing variety of architectures and topologies (Hong, 2007; Thallapally *et al.*, 2008). The synthesis of such species is often based on the self-assembly of suitable building blocks to give supramolecular networks constructed by coordination or/and hydrogen bonds or other weaker supromolecular interactions, such as π-π stacking interactions. As a building block, the pyridine-2,6-dicarboxylic acid is a good ligand with multifunctional coordination sites providing intriguing architectures and topologies (Fernandes *et al.*, 2001; Rafizadeh & Amani, 2006; Huang *et al.*, 2008; Idrees *et al.*, 2009). Recently, we obtained the title coordination polymer, which was synthesized under hydrothermal conditions.

In the structure of the title compound (Fig. 1), one of the ligands is fully deprotonated while the second carries an OH group. Each Dy^III^ centre is eight -coordinated by six oxygen atoms two N atoms from two pyridine-2,6-dicarboxylato  and two  6-carboxypicolinato ligands, and can described as having a bicapped trigonal prismatic geometry with Dy—O distances and O—Dy—O angles ranging from 2.266 (2) Å to 2.513 (2) Å (Table 1) and 76.75.30 (8) %A to 153.60 (9) %A, respectively. It is of interest that the Dy···O and Dy···N distances are slightly longer than the corresponding values in the isostructural Ho complex (Fernandes *et al.*, 2001). Indeed the average Dy—O bond distance is 2.396 Å, some 0.1 Å longer than the corresponding distance (Ho—O = 2.385Å) in the isomorphous holmium complex as anticipated due to the lanthanide contraction.  The pyridine-2,6-dicarboxylato ligands act as bridges linking adjacent Dy^III^ metal centres into a layer parallel to the (1 0 0) plane. Within the layer, the 6-carboxypicolinato ligands are both hydrogen bond donors and hydrogen bond acceptors with O—H···O and C—H···O hydrogen bonding interactions stabilizing the crystal structure (Table 2). π-π stacking interactions (the centroid-centroid distance between neighboring pyridyl rings is 3.827 (3) Å) and C—H···O hydrogen bonding interactions connect those layers to produce a three-dimensional supramolecular motif (Fig. 2). Other π-π stacking interactions between neighboring pyridyl rings are also present in each layer, the centroid-centroid distance is 3.501 (2) Å.

### Experimental

A mixture of Dy_2_O_3_ (0.375 g; 1 mmol), pyridine-2,6-dicarboxylic acid (0.167 g; 1 mmol),  water (10 ml) and HNO_3_ (0.024 g; 0.385 mmol) was stirred vigorously for 20 min and then sealed in a Teflon-lined stainless-steel autoclave (20 ml, capacity). The autoclave was heated and maintained at 433 K for 3 days, and then cooled to room temperature at 5 K h^-1^  to yield colorless block-like crystals.

### Refinement

The H atom of the 6-carboxypicolinate ligand was located in a difference Fourier map and refined with a distance restraint of O–H = 0.82 Å, and with *U*_iso_(H) = 1.5 *U*_eq_(O). H atoms attached to C were placed at calculated positions and were treated as riding on their parent atoms with C—H = 0.93 Å, and with *U*_iso_(H) = 1.2 *U*_eq_(C).

### Crystal data

**Table:**

[Dy(C_7_H_3_NO_4_)(C_7_H_4_NO_4_)]	*F*(000) = 940
*M**_r_* = 493.72	*D*_x_ = 2.437 Mg m^−^^3^
Monoclinic, *P*2_1_/*c*	Mo *K*α radiation, λ = 0.71073 Å
Hall symbol: -P 2ybc	Cell parameters from 5626 reflections
*a* = 12.2151 (14) Å	θ = 2.4–27.8°
*b* = 8.3703 (10) Å	µ = 5.61 mm^−^^1^
*c* = 13.4698 (16) Å	*T* = 296 K
β = 102.332 (1)°	Block, colourless
*V* = 1345.4 (3) Å^3^	0.23 × 0.21 × 0.19 mm
*Z* = 4	

### Data collection

**Table:**

Bruker APEXII area-detector diffractometer	2413 independent reflections
Radiation source: fine-focus sealed tube	2305 reflections with *I* > 2σ(*I*)
graphite	*R*_int_ = 0.024
φ and ω scans	θ_max_ = 25.2°, θ_min_ = 1.7°
Absorption correction: multi-scan (*SADABS*; Bruker, 2004)	*h* = −14→12
*T*_min_ = 0.359, *T*_max_ = 0.415	*k* = −8→10
6670 measured reflections	*l* = −16→16

### Refinement

**Table:**

Refinement on *F*^2^	Primary atom site location: structure-invariant direct methods
Least-squares matrix: full	Secondary atom site location: difference Fourier map
*R*[*F*^2^ > 2σ(*F*^2^)] = 0.021	Hydrogen site location: inferred from neighbouring sites
*wR*(*F*^2^) = 0.051	H atoms treated by a mixture of independent and constrained refinement
*S* = 1.12	*w* = 1/[σ^2^(*F*_o_^2^) + (0.0241*P*)^2^ + 1.92*P*] where *P* = (*F*_o_^2^ + 2*F*_c_^2^)/3
2413 reflections	(Δ/σ)_max_ = 0.002
229 parameters	Δρ_max_ = 0.58 e Å^−^^3^
1 restraint	Δρ_min_ = −1.43 e Å^−^^3^

### Special details

**Table:**

Geometry. All esds (except the esd in the dihedral angle between two l.s. planes) are estimated using the full covariance matrix. The cell esds are taken into account individually in the estimation of esds in distances, angles and torsion angles; correlations between esds in cell parameters are only used when they are defined by crystal symmetry. An approximate (isotropic) treatment of cell esds is used for estimating esds involving l.s. planes.
Refinement. Refinement of F^2^ against ALL reflections. The weighted R-factor wR and goodness of fit S are based on F^2^, conventional R-factors R are based on F, with F set to zero for negative F^2^. The threshold expression of F^2^ > 2sigma(F^2^) is used only for calculating R-factors(gt) etc. and is not relevant to the choice of reflections for refinement. R-factors based on F^2^ are statistically about twice as large as those based on F, and R- factors based on ALL data will be even larger.

### Fractional atomic coordinates and isotropic or equivalent isotropic displacement parameters (Å^2^)

**Table:**

	*x*	*y*	*z*	*U*_iso_*/*U*_eq_	
Dy1	0.197015 (13)	0.232131 (18)	0.818183 (11)	0.00903 (7)	
C2	0.5158 (3)	0.5574 (5)	0.8746 (3)	0.0243 (9)	
H2	0.5247	0.6677	0.8750	0.029*	
C3	0.4095 (3)	0.4901 (4)	0.8567 (3)	0.0184 (8)	
C4	0.4836 (3)	0.2361 (4)	0.8735 (3)	0.0187 (8)	
C1	0.6084 (3)	0.4572 (5)	0.8919 (3)	0.0282 (9)	
H1	0.6805	0.4990	0.9030	0.034*	
C5	0.5912 (3)	0.2933 (5)	0.8922 (3)	0.0274 (9)	
H5	0.6518	0.2233	0.9049	0.033*	
N1	0.3933 (2)	0.3321 (3)	0.8555 (2)	0.0136 (6)	
C6	0.3019 (3)	0.5872 (4)	0.8367 (3)	0.0201 (8)	
O1	0.2131 (2)	0.5091 (3)	0.82690 (18)	0.0180 (6)	
O2	0.3085 (3)	0.7328 (3)	0.8293 (3)	0.0452 (10)	
C7	0.4611 (3)	0.0608 (4)	0.8716 (3)	0.0203 (8)	
O4	0.3508 (2)	0.0270 (3)	0.8468 (2)	0.0202 (6)	
O3	0.5333 (2)	−0.0385 (3)	0.8889 (3)	0.0368 (8)	
C13	0.2072 (3)	0.1845 (4)	1.0625 (2)	0.0128 (7)	
C8	0.1581 (3)	0.0254 (4)	1.0222 (2)	0.0122 (7)	
O5	0.2310 (2)	0.2782 (3)	0.9969 (2)	0.0200 (6)	
N2	0.1355 (2)	0.0186 (3)	0.9205 (2)	0.0107 (6)	
C11	0.0664 (3)	−0.2478 (4)	0.9275 (3)	0.0171 (8)	
H11	0.0339	−0.3386	0.8937	0.021*	
C12	0.0876 (3)	−0.1133 (4)	0.8745 (2)	0.0115 (7)	
O7	0.1092 (2)	0.0071 (3)	0.72203 (17)	0.0148 (5)	
O6	0.2198 (2)	0.2145 (3)	1.15526 (19)	0.0184 (6)	
C14	0.0590 (3)	−0.0988 (4)	0.7607 (2)	0.0115 (7)	
C9	0.1386 (3)	−0.1020 (4)	1.0812 (3)	0.0152 (7)	
H9	0.1539	−0.0945	1.1517	0.018*	
C10	0.0953 (3)	−0.2420 (4)	1.0324 (3)	0.0183 (8)	
H10	0.0858	−0.3317	1.0703	0.022*	
O8	−0.0153 (2)	−0.1898 (3)	0.71176 (18)	0.0171 (5)	
H2A	0.334 (3)	0.822 (3)	0.838 (3)	0.026*	

### Atomic displacement parameters (Å^2^)

**Table:**

	*U*^11^	*U*^22^	*U*^33^	*U*^12^	*U*^13^	*U*^23^
Dy1	0.00974 (11)	0.00762 (10)	0.01008 (11)	−0.00032 (5)	0.00291 (7)	0.00052 (5)
C2	0.026 (2)	0.0179 (19)	0.030 (2)	−0.0111 (16)	0.0069 (17)	−0.0045 (16)
C3	0.022 (2)	0.0131 (17)	0.0203 (19)	−0.0011 (15)	0.0048 (15)	0.0002 (14)
C4	0.015 (2)	0.0168 (18)	0.024 (2)	−0.0005 (14)	0.0039 (16)	−0.0011 (14)
C1	0.019 (2)	0.025 (2)	0.040 (3)	−0.0099 (16)	0.0056 (18)	−0.0064 (17)
C5	0.015 (2)	0.023 (2)	0.042 (3)	−0.0006 (16)	0.0021 (18)	−0.0035 (18)
N1	0.0122 (15)	0.0120 (14)	0.0171 (15)	−0.0013 (12)	0.0043 (12)	0.0005 (11)
C6	0.025 (2)	0.0145 (18)	0.0207 (19)	0.0017 (15)	0.0048 (16)	0.0005 (14)
O1	0.0183 (14)	0.0111 (13)	0.0251 (14)	−0.0001 (10)	0.0057 (11)	0.0010 (9)
O2	0.039 (2)	0.0065 (14)	0.088 (3)	−0.0048 (12)	0.009 (2)	0.0015 (15)
C7	0.016 (2)	0.0165 (18)	0.029 (2)	0.0006 (15)	0.0053 (16)	−0.0004 (15)
O4	0.0121 (13)	0.0105 (12)	0.0378 (16)	−0.0023 (10)	0.0048 (11)	0.0001 (10)
O3	0.0185 (15)	0.0197 (15)	0.070 (2)	0.0055 (12)	0.0043 (15)	0.0011 (14)
C13	0.0114 (17)	0.0152 (17)	0.0122 (18)	0.0025 (13)	0.0031 (14)	0.0011 (13)
C8	0.0097 (17)	0.0157 (17)	0.0124 (17)	0.0037 (13)	0.0049 (13)	−0.0021 (13)
O5	0.0295 (16)	0.0178 (13)	0.0137 (14)	−0.0094 (11)	0.0066 (12)	−0.0016 (10)
N2	0.0102 (14)	0.0123 (14)	0.0102 (14)	−0.0009 (11)	0.0036 (11)	−0.0004 (11)
C11	0.024 (2)	0.0125 (17)	0.017 (2)	−0.0039 (13)	0.0096 (17)	−0.0024 (13)
C12	0.0103 (17)	0.0112 (16)	0.0143 (17)	−0.0011 (13)	0.0053 (13)	0.0000 (13)
O7	0.0177 (13)	0.0146 (12)	0.0131 (12)	−0.0036 (10)	0.0054 (10)	−0.0004 (9)
O6	0.0262 (15)	0.0182 (13)	0.0119 (13)	−0.0017 (11)	0.0064 (11)	−0.0037 (10)
C14	0.0093 (17)	0.0120 (16)	0.0139 (17)	0.0019 (13)	0.0043 (13)	−0.0033 (13)
C9	0.0166 (18)	0.0191 (18)	0.0107 (17)	0.0001 (14)	0.0044 (14)	0.0015 (13)
C10	0.019 (2)	0.0191 (18)	0.018 (2)	−0.0012 (14)	0.0068 (16)	0.0061 (13)
O8	0.0159 (13)	0.0198 (13)	0.0163 (13)	−0.0061 (11)	0.0052 (10)	−0.0045 (10)

### Geometric parameters (Å, °)

**Table:**

Dy1—O8^i^	2.266 (2)	O2—H2A	0.811 (19)
Dy1—O6^ii^	2.314 (3)	C7—O3	1.198 (5)
Dy1—O1	2.328 (2)	C7—O4	1.347 (4)
Dy1—O5	2.385 (3)	C13—O6	1.251 (4)
Dy1—O7	2.405 (2)	C13—O5	1.261 (4)
Dy1—N2	2.469 (3)	C13—C8	1.513 (5)
Dy1—N1	2.488 (3)	C8—N2	1.341 (4)
Dy1—O4	2.513 (2)	C8—C9	1.381 (5)
C2—C1	1.388 (6)	N2—C12	1.338 (4)
C2—C3	1.389 (6)	C11—C10	1.382 (6)
C2—H2	0.9300	C11—C12	1.387 (5)
C3—N1	1.336 (4)	C11—H11	0.9300
C3—C6	1.519 (5)	C12—C14	1.502 (5)
C4—N1	1.344 (5)	O7—C14	1.253 (4)
C4—C5	1.371 (6)	O6—Dy1^iii^	2.314 (3)
C4—C7	1.492 (5)	C14—O8	1.257 (4)
C1—C5	1.388 (6)	C9—C10	1.392 (5)
C1—H1	0.9300	C9—H9	0.9300
C5—H5	0.9300	C10—H10	0.9300
C6—O2	1.226 (4)	O8—Dy1^iv^	2.266 (2)
C6—O1	1.250 (5)		
			
O8^i^—Dy1—O6^ii^	95.01 (9)	C1—C5—H5	120.5
O8^i^—Dy1—O1	77.91 (9)	C3—N1—C4	118.4 (3)
O6^ii^—Dy1—O1	80.18 (9)	C3—N1—Dy1	117.9 (2)
O8^i^—Dy1—O5	94.91 (9)	C4—N1—Dy1	123.6 (2)
O6^ii^—Dy1—O5	153.60 (9)	O2—C6—O1	125.5 (4)
O1—Dy1—O5	78.08 (8)	O2—C6—C3	118.5 (4)
O8^i^—Dy1—O7	79.79 (9)	O1—C6—C3	115.9 (3)
O6^ii^—Dy1—O7	76.74 (8)	C6—O1—Dy1	126.0 (2)
O1—Dy1—O7	146.15 (8)	C6—O2—H2A	159 (3)
O5—Dy1—O7	129.18 (8)	O3—C7—O4	124.0 (3)
O8^i^—Dy1—N2	84.53 (9)	O3—C7—C4	123.6 (4)
O6^ii^—Dy1—N2	141.40 (9)	O4—C7—C4	112.4 (3)
O1—Dy1—N2	136.47 (9)	C7—O4—Dy1	124.8 (2)
O5—Dy1—N2	64.02 (8)	O6—C13—O5	125.3 (3)
O7—Dy1—N2	65.15 (8)	O6—C13—C8	119.3 (3)
O8^i^—Dy1—N1	143.55 (10)	O5—C13—C8	115.4 (3)
O6^ii^—Dy1—N1	79.66 (9)	N2—C8—C9	122.2 (3)
O1—Dy1—N1	65.64 (9)	N2—C8—C13	112.5 (3)
O5—Dy1—N1	77.84 (10)	C9—C8—C13	125.3 (3)
O7—Dy1—N1	132.22 (9)	C13—O5—Dy1	126.2 (2)
N2—Dy1—N1	121.72 (9)	C12—N2—C8	118.8 (3)
O8^i^—Dy1—O4	153.64 (9)	C12—N2—Dy1	119.6 (2)
O6^ii^—Dy1—O4	92.28 (9)	C8—N2—Dy1	121.3 (2)
O1—Dy1—O4	128.35 (8)	C10—C11—C12	117.6 (3)
O5—Dy1—O4	89.58 (9)	C10—C11—H11	121.2
O7—Dy1—O4	77.30 (8)	C12—C11—H11	121.2
N2—Dy1—O4	74.15 (9)	N2—C12—C11	122.9 (3)
N1—Dy1—O4	62.74 (8)	N2—C12—C14	112.9 (3)
C1—C2—C3	118.9 (3)	C11—C12—C14	124.2 (3)
C1—C2—H2	120.6	C14—O7—Dy1	122.1 (2)
C3—C2—H2	120.6	C13—O6—Dy1^iii^	166.3 (2)
N1—C3—C2	122.2 (3)	O7—C14—O8	125.0 (3)
N1—C3—C6	114.1 (3)	O7—C14—C12	116.9 (3)
C2—C3—C6	123.7 (3)	O8—C14—C12	118.1 (3)
N1—C4—C5	122.8 (3)	C8—C9—C10	118.2 (3)
N1—C4—C7	116.3 (3)	C8—C9—H9	120.9
C5—C4—C7	120.8 (3)	C10—C9—H9	120.9
C2—C1—C5	118.7 (4)	C11—C10—C9	120.1 (3)
C2—C1—H1	120.7	C11—C10—H10	120.0
C5—C1—H1	120.7	C9—C10—H10	120.0
C4—C5—C1	119.0 (4)	C14—O8—Dy1^iv^	146.8 (2)
C4—C5—H5	120.5		

Symmetry codes: (i) −*x*, *y*+1/2, −*z*+3/2; (ii) *x*, −*y*+1/2, *z*−1/2; (iii) *x*, −*y*+1/2, *z*+1/2; (iv) −*x*, *y*−1/2, −*z*+3/2.

### Hydrogen-bond geometry (Å, °)

**Table:**

*D*—H···*A*	*D*—H	H···*A*	*D*···*A*	*D*—H···*A*
O2—H2A···O4^v^	0.81 (2)	1.73 (2)	2.518 (3)	164 (5)
O2—H2A···O3^v^	0.81 (2)	2.65 (3)	3.303 (4)	139 (4)
C9—H9···O1^iii^	0.93	2.42	3.332 (4)	165
C1—H1···O5^vi^	0.93	2.42	3.123 (5)	133
C2—H2···O3^v^	0.93	2.47	3.393 (5)	174

Symmetry codes: (v) *x*, *y*+1, *z*; (iii) *x*, −*y*+1/2, *z*+1/2; (vi) −*x*+1, −*y*+1, −*z*+2.
